# Effects of Acute Cold Stress on Liver *O*-GlcNAcylation and Glycometabolism in Mice

**DOI:** 10.3390/ijms19092815

**Published:** 2018-09-18

**Authors:** Ruizhi Yao, Yuying Yang, Shuai Lian, Hongzhao Shi, Peng Liu, Yang Liu, Huanmin Yang, Shize Li

**Affiliations:** College of Animal Science and Veterinary Medicine, Heilongjiang Bayi Agricultural University, Daqing 163319, China; 15848955365@163.com (R.Y.); yalele258@sina.com (Y.Y.); lianlianshuai@163.com (S.L.); 15776557846@163.com (H.S.); 18346663533@163.com (P.L.); lymissive@163.com (Y.L.); byndyhm@163.com (H.Y.)

**Keywords:** *O*-GlcNAc, cold stress, glucose metabolism, autophagy, apoptosis

## Abstract

Protein *O*-linked β-*N*-acetylglucosamine glycosylation (*O*-GlcNAcylation) regulates many biological processes. Studies have shown that *O*-GlcNAc modification levels can increase during acute stress and suggested that this may contribute to the survival of the cell. This study investigated the possible effects of *O*-GlcNAcylation that regulate glucose metabolism, apoptosis, and autophagy in the liver after acute cold stress. Male C57BL/6 mice were exposed to cold conditions (4 °C) for 0, 2, 4, and 6 h, then their livers were extracted and the expression of proteins involved in glucose metabolism, apoptosis, and autophagy was determined. It was found that acute cold stress increased global *O*-GlcNAcylation and protein kinase B (AKT) phosphorylation levels. This was accompanied by significantly increased activation levels of the glucose metabolism regulators 160 kDa AKT substrate (AS160), 6-phosphofructo-2-kinase/fructose-2,6-biphosphatase 2 (PFKFB2), and glycogen synthase kinase-3β (GSK3β). The levels of glycolytic intermediates, fructose-1,6-diphosphate (FDP) and pyruvic acid (PA), were found to show a brief increase followed by a sharp decrease. Additionally, adenosine triphosphate (ATP), as the main cellular energy source, had a sharp increase. Furthermore, the B-cell lymphoma 2(Bcl-2)/Bcl-2-associated X (Bax) ratio was found to increase, whereas cysteine-aspartic acid protease 3 (caspase-3) and light chain 3-II (LC3-II) levels were reduced after acute cold stress. Therefore, acute cold stress was found to increase *O*-GlcNAc modification levels, which may have resulted in the decrease of the essential processes of apoptosis and autophagy, promoting cell survival, while altering glycose transport, glycogen synthesis, and glycolysis in the liver.

## 1. Introduction

Stress is known to affect multiple biochemical regulatory systems, and is considered to be a major risk factor for a number of different diseases [1]. Stress has been defined as the non-specific response of an organism to varying environmental demands [2]. Organisms are regularly exposed to unfavorable environmental conditions, including cold, heat, and UV stress. In particular, cold stress has a wide range of effects on many systems of the organism, including the neuroendocrine [3], reproductive [4], and cardiovascular [5] systems. Additionally, cold stress also has a significant impact on the organism’s energy metabolism [6], immune function [7], and antioxidant function [8].

Recently, *O*-linked β-*N*-acetylglucosamine (*O*-GlcNAc) modification levels have been considered a real-time measure of cellular nutrient status, with the levels of *O*-GlcNAc modification increasing in response to elevated nutrient and energy availabilities [9]. Studies have shown that dynamic *O*-linked β-*N*-acetylglucosamine glycosylation (*O*-GlcNAcylation) of nuclear, cytoplasmic, and mitochondrial proteins regulates more than 4000 proteins in a pattern similar to protein phosphorylation [10,11]. *O*-GlcNAc is widely expressed on serine and threonine residues of proteins, and there are two enzymes, *O*-GlcNAc transferase (OGT) and *O*-GlcNAcase (OGA), known to mediate the addition and removal of *O*-GlcNAc, respectively [12]. *O*-GlcNAcylation has been implicated in the regulation of signal transduction, gene expression, transcription, cell cycle, mitochondrial motility, circadian clock, autophagosome maturation, proteasomal degradation, nutrient sensing, and cellular stress response [13,14,15,16,17,18,19,20,21,22]. Specifically, in the state of inflammation and during stress, *O*-GlcNAc can act as a “stress sensor” [23,24]. *O*-GlcNAc can protect the organism by inhibiting inflammation and apoptosis, while also reducing protein degradation and regulating cell metabolism. Additionally, studies have shown that when the cell feels stress, the global *O*-GlcNAc modification level increases, along with the tolerance and survival of the cell [25]. Furthermore, studies have shown that glycosylation of proteins with *O*-GlcNAc is also involved in the regulation of energy metabolism, autophagy, and apoptosis [21,26,27].

Glycogen, a polymer of glucose residues, is considered an important form of energy storage, particularly in the liver [28]. In the liver, glycogen synthesis and degradation play major roles in regulating blood glucose homeostasis as well as supplying energy to other tissues [29,30]. *O*-GlcNAc signaling is known to play a key role in many aspects of liver metabolism, including insulin sensitivity, glycose transport, glycogen synthesis, gluconeogenesis, and lipogenesis [26].

Autophagy is a regulated nutrient recycling pathway that can affect both cell survival and cell death [31]. Normal physiological levels of autophagy promote cellular survival in response to a variety of stress conditions, including starvation, hypoxia, mitochondrial damage, and pathogen infection [32]. It has been reported that increased cardiac *O*-GlcNAcylation leads to blocked autophagic signaling in the diabetic heart [33]. Furthermore, a protein regulating autophagosome-lysosome fusion, SNAP-29, is *O*-GlcNAcylated and promotes autophagy, by decreasing its *O*-GlcNAc modification [21]. However, additional research on the effects of *O*-GlcNAc modification under acute cold stress on hepatic metabolism and autophagy in mice is limited. Therefore, this study focused on the role of *O*-GlcNAc modification in the liver of mice after acute cold stress.

## 2. Results

### 2.1. Time-Course of Plasma Glucose, Insulin, and Glucagon after Acute Cold Stress

**Figure 1:**
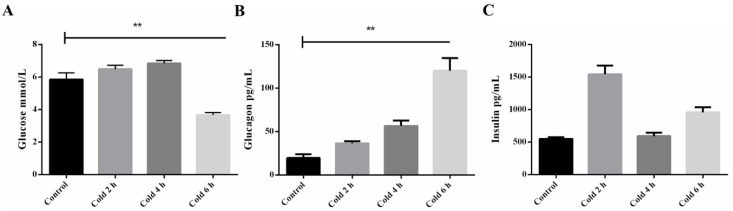
Effect of acute cold stress on the levels of (**A**) plasma glucose, (**B**) glucagon, and (**C**) insulin in the liver. (**A**) The data are presented as mean ± standard error of the mean (SEM) (*n* = 8). Statistically significant differences are indicated, ** *p* < 0.01.

To test changes in glucose, insulin, and glucagon under acute cold stress in plasma, the authors of this study examined and compared the response of both the control and stressed groups. Glycemia levels at 6 h were found to be significantly different between the control and stressed groups (see Figure 1A) (*p* < 0.05). There are a variety of hormones in the body that regulate blood sugar levels including insulin and glucagon. Therefore, the authors investigated whether changes in insulin and glucagon levels occurred during acute cold stimulation. As shown in Figure 1B, an increase in glucagon levels was observed in the cold stimulation group compared to the control group, with a significant difference noted at 6 h (*p* < 0.05). However, insulin levels showed a fluctuation regulation after acute cold stimulation (see Figure 1C).

### 2.2. Acute Cold Stress Induced Changes to Fructose-1,6-diphosphate (FDP) and Pyruvic Acid (PA) in the Liver

**Figure 2:**
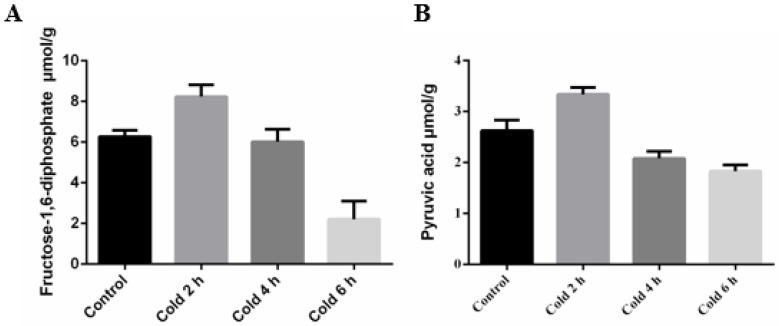
Effect of acute cold stress on the levels of glycolytic intermediates (**A**) fructose-1,6-diphosphate (FDP) and (**B**) pyruvic acid (PA) in the liver. The data are presented as mean ± SEM (*n* = 8). Statistically significant differences are indicated.

In order to test changes to glycolysis in the liver, the authors examined the glycolytic high-energy intermediate fructose-1,6-diphosphate (FDP) and the final product pyruvic acid (PA). It was found that acute cold stress caused a significant change to the levels of both FDP and PA in the liver of mice after 6 h (see Figure 2A,B, respectively).

### 2.3. Adenosine Triphosphate (ATP) Levels in the Liver Were Affected by Acute Cold Stress

**Figure 3:**
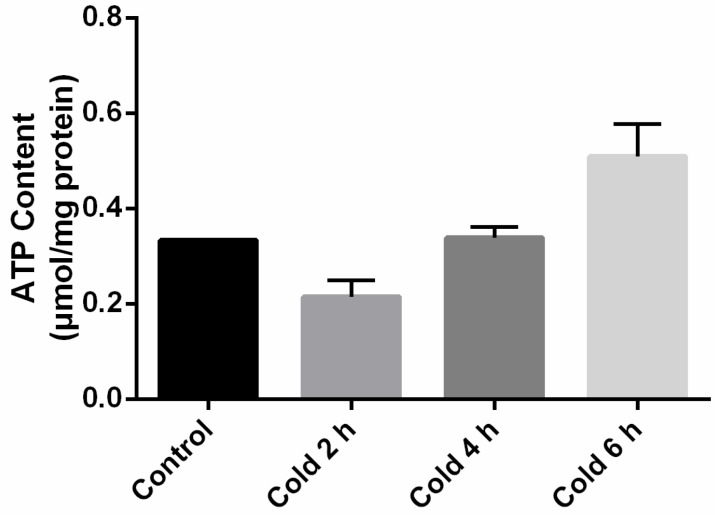
Effect of acute cold stress on the levels of adenosine triphosphate (ATP) in the liver. The data are presented as mean ± SEM (*n* = 8). Statistically significant differences are indicated.

Cold stress causes the organism to produce more energy to ensure that energetic balance is maintained. The rapid consumption of glycogen suggests that the liver is trying to maintain the energetic balance through a metabolic response. To determine the energetic status of liver cells from mice during acute cold exposure, the authors measured the intracellular adenosine triphosphate (ATP) levels. As shown in Figure 3, acute cold exposure caused a transient decrease in ATP, which was followed by a prolonged increase in the adenosine triphosphate ATP levels, with a significant difference seen after 6 h.

### 2.4. Global Levels of O-GlcNAc Glycosylation Increase after Acute Cold Stress

**Figure 4:**
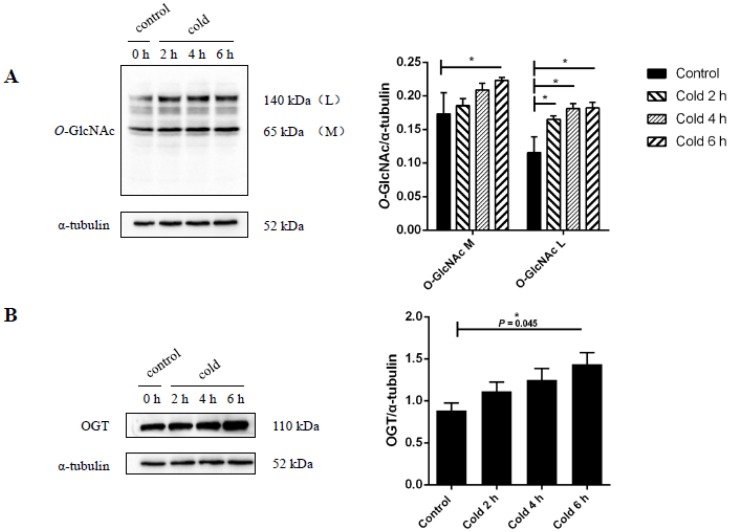
Effect of acute cold stress on the expression of global levels of *O*-linked β-*N*-acetylglucosamine (*O*-GlcNAc) glycosylation and *O*-GlcNAc transferase (OGT) protein in the liver. (**A**) Global *O*-GlcNAcylation levels in liver and (**B**) levels of OGT. The data are presented as mean ± SEM (*n* = 8). Statistically significant differences are indicated, * *p* < 0.05.

It was found that cold stress resulted in increased expression of global *O*-GlcNAc glycosylation levels over time (see Figure 4A). *O*-GlcNAcylation levels at 6 h were found to be significantly different between the stressed and control groups (*p* < 0.05). This global increase in *O*-GlcNAcylation levels would require the activation of the transferase, OGT. Therefore, the authors investigated whether cold stimulation was accompanied by changes to OGT expression. To assess the expression levels of OGT, immunoblots were performed on liver tissue lysates from the control and cold stimulation groups. As shown in Figure 4B, an increase in OGT expression was observed in the cold stimulation group compared to the control group, with the intensity of the OGT band found to be significantly different at 6 h (*p* < 0.05).

### 2.5. Cold Stress Induces Protein Kinase B (AKT) Activation and Promotes Glucose Metabolism

**Figure 5:**
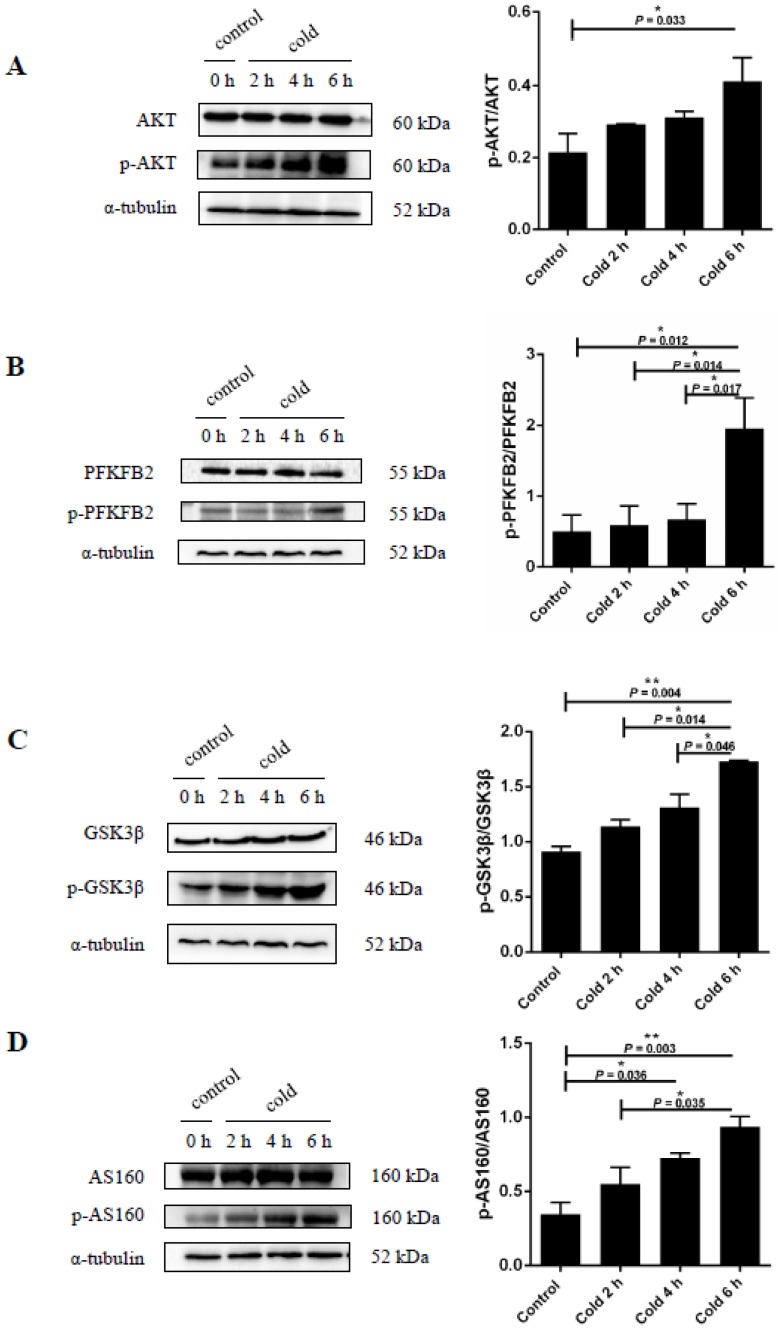
Effect of acute cold stress on glucose metabolism in the liver. (**A**) Protein kinase B (AKT) protein levels and phosphorylation state in the liver; (**B**) 6-phosphofructo-2-kinase/fructose-2,6-biphosphatase 2 (PFKFB2) protein levels and phosphorylation state in the liver; (**C**) glycogen synthase kinase-3β (GSK3β) protein levels and phosphorylation state in the liver; (**D**) 160 kDa AKT substrate (AS160) protein levels and phosphorylation state in the liver. The data are presented as mean ± SEM (*n* = 8). Statistically significant differences are indicated, * *p* < 0.05 and ** *p* < 0.01.

When the body is unable to use glucose from food, hepatic glycogen becomes an important source of glucose. Therefore, in order to study the effect of cold stress on glucose metabolism in the body, mice were exposed to cold stimulation at 4 °C while fasting. This was followed by the levels of expression of protein kinase B (AKT) signaling proteins in the liver analyzed by immunoblots. It was found that cold stress increased AKT phosphorylation at a particular residue, Ser^473^ (see Figure 5A). Additionally, after 6 h of cold stress AKT phosphorylation levels were found to be significantly increased (*p* < 0.05). Since AKT activation is known to be involved in glucose uptake and consumption [34], glucose metabolism-related enzymes were also measured. As seen in Figure 5B–D, cold stress was found to affect the phosphorylation levels of the glucose metabolism enzymes GSK3β, PFKFB2, and AS160. The expression of p-AS160 (TBC1D4) (Thr^642^), p-GSK3β (Ser^9^), and p-PFKFB2 (Ser^483^) were significantly increased after exposure to the cold for 6 h compared to the control group (*p* < 0.05).

### 2.6. Effect of Cold Stress on Bcl-2/Bax Ratio and Caspase-3 Protein Expression

**Figure 6:**
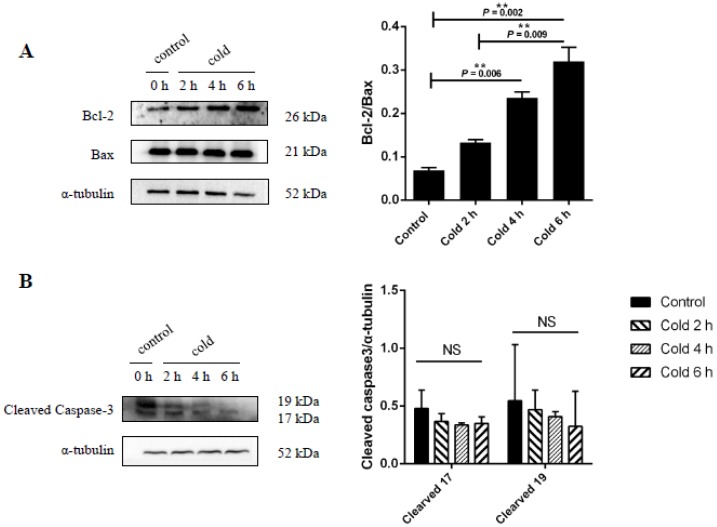
Effect of acute cold stress on the expression of (**A**) Bcl-2, Bax, and (**B**) caspase-3 proteins in the liver. The data are presented as mean ± SEM (*n* = 8). Statistically significant differences are indicated, ** *p* < 0.01. NS: not significant.

To determine the influence of cold stress on cell apoptosis, the expression of Bcl-2, Bax, and caspase-3 were measured in the liver after exposure to cold conditions. As shown in Figure 6A, the expression of the Bcl-2/Bax after 6 h was found to be significantly increased in the stressed group compared to the control group (*p* < 0.05). In addition, the expression of caspase-3 was found to decrease after cold stress; however, this change was not significant when compared to the control group (see Figure 6B).

### 2.7. Cold Stress Promotes Autophagy by Activating the AKT/mTOR Pathway

**Figure 7:**
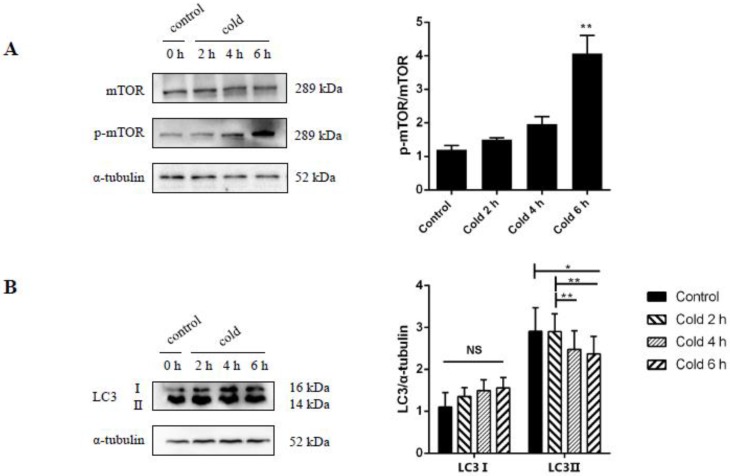
Effect of acute cold stress on the expression of mammalian target of (**A**) rapamycin (mTOR) phosphorylation and (**B**) LC3-II proteins in the liver. The data are presented as mean ± SEM (*n* = 8). Statistically significant differences are indicated, * *p* < 0.05 and ** *p* < 0.01. NS: not significant.

To determine the influence of acute cold stress on autophagy, an analysis of autophagy markers such as AKT, mammalian target of rapamycin (mTOR) complex, and chain 3-II (LC3-II) was performed. It was further confirmed that acute cold stress has a marked effect on AKT activity as shown by the previous western blot analysis, with the phosphorylation of AKT at Ser^473^ (see Figure 5A). mTOR is a classic pathway in autophagy and, by western blot analysis, it was found that the mTOR signaling was suppressed by significantly increased phosphorylation of mTOR at Ser^2448^ (see Figure 7A, *p* < 0.05). Furthermore, under cold stress the conversion of LC3-I to LC3-II was significantly decreased, suggesting that cold stress has an inhibitory effect on autophagic activity (see Figure 7B, *p* < 0.05).

## 3. Discussion

An efficient cold defense mechanism requires an accurate coordination of energy sources (e.g., glucose and fatty acids) and heat production (e.g., shivering and adaptive thermogenesis) [35]. Cold exposure increases food intake, leading to exogenous glucose supplementation in mice, which may have an effect on hepatic glucose metabolism [35]. In this study, the authors performed fasting treatment on experimental mice during cold exposure to avoid the effect of exogenous glucose. The liver, as the center of intermediary metabolism, releases glucose into the circulation during acute cold exposure to meet the energy demands [36]. It has been seen that all of the tissues except the liver increase their glucose uptake after cold exposure [37]. Additionally, previous studies have demonstrated that 4 h of exposure to −8 °C depleted liver glycogen, and caused liver damage in rats [38]. In this study, it was demonstrated that acute cold stimulation led to a decrease of plasma glucose levels, providing further evidence that cold stress increases glucose utilization. There are a variety of hormones in the organism that regulate plasma glucose levels, including insulin and glucagon. The results of this study indicated that cold exposure could increase plasma levels of glucagon, with corresponding changes in insulin levels also observed. Insulin is one of the most important hormones in the body. Its secretion has been precisely regulated by various substances such as nutrients, neurotransmitters, and hormones [39,40,41]. Research has shown that a key role for the brain is to act, via the sympathetic nervous system, in the rapid, highly coordinated, and reciprocal changes of insulin secretion and insulin sensitivity that preserve glucose homeostasis in a setting of cold exposure [42]. In this study, insulin levels were found to change irregularly, perhaps due to some negative feedback regulation caused by a nervous activity disorder to ensure blood glucose homeostasis, which is subject to further verification.

Stress due to either environmental or internal conditions induces signal transduction that ultimately leads to the production and activation of proteins that weaken the effects of harmful cellular conditions, and counteract the signals that promote apoptosis [43]. Protein phosphorylation and *O*-GlcNAcylation are associated with these signal transduction pathways [44]. It has been shown previously that *O*-GlcNAc signaling is an essential stress and metabolic sensor, and that global *O*-GlcNAcylation levels and OGT activity are modulated by various stress stimuli [45]. Acute oxidative stress can induce both an increase and a decrease in global *O*-GlcNAcylation, thereby regulating many cellular pathways to promote cell and tissue survival [46]. Furthermore, enhanced global *O*-GlcNAc signaling is known to reduce endoplasmic reticulum (ER) stress-induced cell death [47]. Additionally, it has been reported that increased global *O*-GlcNAcylation levels in heat stress models promote the induction of heat shock proteins and cell survival [44]. Taken together, there are multiple lines of evidence that suggest that an increase of *O*-GlcNAc modification may be protective of the cell in response to stress [44]. In almost all of the mammalian cell/tissue types examined so far, one of the earliest responses to cellular stress is the elevation of *O*-GlcNAcylation of many proteins [48]. In this study, the authors demonstrated the effect of 4 °C after 0, 2, 4, and 6 h of treatment on the regulation of the *O*-GlcNAc and OGT dynamics in C57BL/6 liver tissue. It was found that acute cold stress caused global *O*-GlcNAc glycosylation levels to rise, highlighting the response of the mice to ensure self-protection by promoting cell survival. The changes in the expression of Bcl-2/Bax further confirmed the pro-survival effect of *O*-GlcNAcylation modification.

Apoptosis, a process of programmed cell death, is regulated by many molecular signaling pathways, including the mitochondrial pathway [49,50]. In these pathways, mitochondrial outer membrane permeabilization is tightly controlled by the Bcl-2 family of proteins [51]. Bcl-2 is an anti-apoptotic protein that maintains the integrity of the mitochondrial membrane, whereas Bax is a pro-apoptotic protein that can destroy the integrity of the membrane [52]. *O*-GlcNAcylation plays a fundamental role in regulatory mechanisms by modifying proteins involved in cell signaling and apoptosis [27]. Previously, it has been shown that in primary cultures of neonatal rat ventricular myocytes, increased *O*-GlcNAcylation levels altered the expression and translocation of members of the Bcl-2 protein family, promoting cell survival [53]. In this study, the expression levels of cleaved caspase-3, Bcl-2, and Bax were analyzed by western blot. The results showed that acute cold stress decreased cleaved caspase-3 levels, and increased Bcl-2 levels; however, there were no effects on Bax levels. These findings suggest that *O*-GlcNAc glycosylation may protect the liver against hepatocyte apoptosis during acute cold stress.

In addition, studies have shown that *O*-GlcNAc also plays an important role in the regulation of autophagy. Autophagy is a highly conserved endogenous cell survival mechanism that is typically activated in response to external stress [33]. During cellular conditions of stress and starvation, activators of autophagy can respond to nutrient, energy, oxygen, and hormonal demands to maintain metabolic homeostasis [54]. A number of studies have shown that the AKT/mTOR pathways play an important role in autophagy regulation, negatively regulating autophagy by inhibiting unc-51-like autophagy activating kinase 1 (ULK1) [55,56,57]. It has been shown previously that elevated protein *O*-GlcNAcylation inhibited autophagy in *Drosophila melanogaster*, whereas suppressing protein *O*-GlcNAcylation enhanced autophagic activity, implying a negative regulatory role of *O*-GlcNAcylation in autophagy [58]. In mammalian HeLa cells treated with the OGT inhibitor, alloxan, *O*-GlcNAcylation was reduced and the promotion of autophagy activity was observed [21], further illustrating the negative regulation of autophagy by *O*-GlcNAcylation. Furthermore, it has been established that the up-regulation of *O*-GlcNAcylation promoted AKT phosphorylation at Ser^473^ [59,60]. In addition, the phosphorylation of AKT activated AKT/mTOR and suppressed autophagy [57]. In this study, autophagy activity was assessed by examining the microtubule-associated protein, LC3-II, by western blot analysis. The results highlighted that acute cold stress induced an increase in the phosphorylated p-AKT (Ser^473^) and p-mTOR (Ser^2448^) levels, and a decrease in the expression of the autophagy-related protein, LC3-II. This suggests that acute cold stress induced the protein *O*-GlcNAcylation, enhancing the inhibition of liver autophagy in mice, consistent with previous research. Many stress pathways sequentially elicit autophagy and apoptosis within the same cell [61]. Generally, autophagy blocks the induction of apoptosis, and apoptosis-associated caspase activation shuts off the autophagic process. However, there are special cases when autophagy or autophagy-relevant proteins may aid in the induction of apoptosis or necrosis, leading to “autophagic cell death” [62]. A previous study has shown that the reduced expression of ATG7 by RNA interference (RNAi) inhibited benzyloxycarbonyl-Val-Ala-Asp-(OMe)-fluoromethyl ketone (zVAD), a caspase inhibitor with broad specificity, and induced cell death [63]. In this study, apoptosis and autophagy were inhibited under acute cold stress conditions. Therefore, it may be hypothesized that this is related to the *O*-GlcNAc self-protection mechanisms. However, how acute cold stress inhibits apoptosis through up-regulating *O*-GlcNAcylation autophagy remains unknown.

Cold stress has been reported to cause profound systemic metabolic changes [64]. In addition to acting as a “stress sensor”, *O*-GlcNAc is proposed to be a “nutrient sensor” to regulate multiple biological pathways [65]. It has been reported that the *O*-GlcNAc modification plays a pivotal role in cold-induced thermogenesis in brown adipose tissue [66]. The liver, an organ that plays a major role in regulating energy availability, has been shown to undergo metabolic changes in response to cold stress by regulating the AKT and Adenosine 5‘-monophosphate (AMP)-activated protein kinase (AMPK) pathways, in order to maintain glucose homeostasis [67]. In the liver of euglycemic mice, changes to the *O*-GlcNAcylation levels have also been shown to affect AKT activity and regulate mRNA expression levels of a key glucose metabolic enzyme, glucose-6-phosphatase [68]. These results have been reinforced in this study, where *O*-GlcNAcylation was increased under acute cold stress, which then promoted AKT activation at Ser^473^. From there, AKT induced the activation of downstream proteins including AS160 (the upstream regulator of glycose transporter), PFKFB2 (the key regulator of glycolysis), and GSK3β (the negative regulator of glycometabolism), as well as regulating glucose metabolism. As a key regulator of glycolysis, PFKFB2 continues to increase in expression during cold exposure. However, cold exposure can lead to a decrease in glycogen and plasma glucose levels in the liver, resulting in a decrease in glycolysis substrates. This leads to a sharp decrease in the levels of glycolytic intermediates FDP and PA. Previously, it has been reported that during fasting the phosphorylation of GSK3β was reduced in the liver [69]. In this study, the mice were fasted during the cold stimulation and exogenous glucose was cut off, but as a negative regulator of glycogen synthesis, the GSK3β phosphorylation levels showed a time-series increase throughout the cold stimulation. GSK3β is a Ser/Thr protein kinase that regulates glycogen metabolism, gene expression, and apoptosis [70]. GSK3β promotes apoptosis through numerous pathways, including acetylation of p53, degradation of Bcl-2, phosphorylation of Bax, and/or phosphorylation of heat shock factor 1 (HSF1) [71]. Furthermore, GSK3β inhibition via phosphorylation of the Ser^9^ residue contributes to the protection needed against oxidative stress and promotes cryoprotection [72].Therefore, it may be hypothesized that the phosphorylation of GSK is related to the self-protection mechanism of GSK3β, which is induced by acute cold stress. Additionally, there is evidence that *O*-GlcNAc can regulate the phosphorylation of GSK3β and thus promote survival, also consistent with the results this study [71]. It has been suggested that upon cold exposure the liver tries to maintain an intracellular energy balance by increasing circulating glucose through the processes of glycogenolysis and gluconeogenesis, and by activating the AKT pathway to promote ATP generation [73]. The level of intracellular ATP of liver cells was found to be significantly reduced after 2 h of cold exposure. This decline seen in the ATP levels is most likely due to the promotion of heat production upon acute cold exposure [74]. Additionally, the main cellular energy source, ATP, is produced primarily from glycolysis and mitochondrial respiration [73]. Furthermore, it has been suggested that the pro-survival Bcl-2 proteins promote mitochondrial respiration [75]. Cellular ATP levels play a pivotal role in cell survival, with the decrease of ATP leading to cell apoptosis [76]. In this study, the authors found that the overall trend of ATP levels in the liver were similar to that of Bcl-2 in hepatocytes. Therefore, it can be speculated that the decrease of hepatocyte apoptosis under acute cold exposure conditions may be related to the change in ATP levels; however, this requires further confirmation.

**Figure 8:**
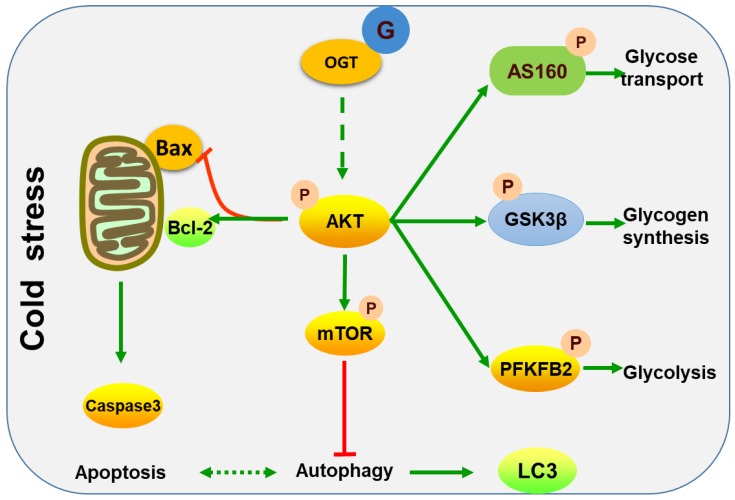
A proposed model for the molecular mechanisms of acute cold stress in the liver. Green solid arrows express promotion, green dotted arrows indicate may promotion, red T-bar represent inhibition.

In summary, this study demonstrated that acute cold stress in mice induced increases in *O*-GlcNAc modification levels, which may be one of the factors resulting in a reduction to apoptosis and autophagy, promoting survival and the regulation of glucose metabolism in the liver. A proposed role of *O*-GlcNAc modification in acute cold stress in the liver is demonstrated in Figure 8. In the future, studies should focus on investigating the influence of *O*-GlcNAc modification in acute cold stress on metabolism and apoptosis, to provide a better understanding of the role of *O*-GlcNAc in acute cold stress.

## 4. Materials and Methods

### 4.1. Antibodies

*O*-GlcNAc (1:1000, #12938S), OGT (1:1000, #23177S), AKT (1:1000, #90272), phospho-AKT (Ser473) (1:1000, #12694), PFKFB2 (1:1000, #13045), phospho-PFKFB2 (Ser483) (1:1000, #13064), phospho-GSK3β (Ser9) (1:1000, #9323), AS160 (1:1000, #2670), phospho-mTOR (Ser2448) (1:1000, #5536), and LC3A/B (1:1000, #12741) antibodies were purchased from CST (Cell Signaling Technology, Danvers, MA, USA). Anti-GSK3 beta (1:8000, #ab32391) and Anti-TBC1D4 (phosphor T642) (1:5000, # ab131214) antibodies were purchased from Abcam (Amyjet Scientific Inc., Upper Heyford, UK). Bax (1:2000, #60267-1-lg), Bcl-2 (1:2000, #60178-1-lg), caspase-3 (1:500, #66470-2), and mTOR (1:1000, #20657-1-AP) antibodies were purchased from Proteintech (Rosemont, IL, USA). Secondary antibodies were labeled with horseradish peroxidase goat anti-mouse IgG (1:20,000, # SA00001-1, Proteintech, USA), goat anti-rabbit IgG (1:20,000, #SA00001-2, Proteintech, USA), and goat anti-mouse IgG, IgM, IgA (1:10,000, ThermoFisher, Munich, Germany, #A-10668).

### 4.2. Animals and Sample Collection

**Figure 9:**
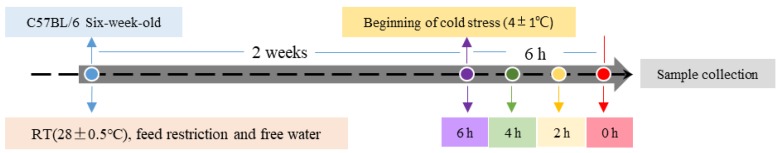
Timeline of the treatment protocol. Mice in the room temperature (RT) control group were left undisturbed, whereas cold mice were exposed to 4 °C.

Six-week-old male pathogen-free C57BL/6 mice were obtained from the Experimental Animal Center of the PLA Academy of Military Medical Sciences (Shenyang, China). The mice were bred and housed in a climatic chamber in which an ambient temperature of 28 ± 0.5 °C and 40% relative humidity were maintained, in a 12 h light/dark cycle (light from 08:00 to 20:00). The mice had restricted feeds (fed from 20:00 to 08:00) and free water. Before being subjected to experimental conditions, the mice were acclimatized for two weeks. After the adaptation period, the mice were randomly allocated into four groups as either control or stressed. The stress conditions were exposed to cold at 4 °C for 2, 4, and 6 h, during fasting and ending at the same time (*n* = 8/each group), whereas the control group (*n* = 8) was maintained at room temperature of 28 ± 0.5 °C. This was followed by simultaneous euthanasia of the control and stress mice groups, and their livers collected. The timeline of the cold stimulation protocol is shown in Figure 9. All procedures were approved by the Institutional Animal Care and Use Committee of the Heilongjiang Bayi Agricultural University (Daqing, China, code, day month year). 

### 4.3. Western Blot Analysis

Tissue samples were lysed in RIPA lysis buffer (#P0013B, Beyotime, Haimen, China) containing phenylmethane sulfonyl fluoride (PMSF) (#ST506, Beyotime, China). Subsequently, tissue samples were homogenized with a ball mill to obtain the total protein lysate. Protein concentrations of the tissue lysates were determined using a BCA protein assay kit (#P0010, Beyotime, China). Equal amounts of protein lysates were then separated by sodium dodecyl sulfate polyacrylamide gel electrophoresis (SDS-PAGE) and transferred to polyvinylidene fluoride (PVDF) membranes (0.45 μm, Millipore, Darmstadt, Germany). PVDF membranes were blocked for 1 h at room temperature with Tris-buffered saline containing 0.05% Tween 20 and 5% powdered non-fat milk, and then incubated overnight with primary antibodies at 4 °C with shaking. Following three 5-min washes with Tris-buffered saline and 0.05% Tween 20, a secondary antibody was added and incubated for 1 h at room temperature. After washing (3 × 5 min), the membranes were visualized by chemiluminescence detection using Luminata Crescendo Western horseradish peroxidase (HRP) substrate (WBLUR0100, Millipore) according to the manufacturer’s instructions. Blots were imaged with the ChemiDoc XRS (Bio-Rad, Hercules, CA, USA) and analyzed using Image J software (http://imagej.nih.gov/ij/).

### 4.4. Measurement of Biochemical Parameters

Serum levels of fasting glucose were measured using commercial cards (IDEXX Laboratories, Westbrook, ME, USA). Serum insulin and glucagon levels were measured by ELISA (Cloud-Clone Corp, Katy, TX, USA). Fructose-1,6-diphosphate (FDP) and pyruvic acid (PA) were measured by ultraviolet spectrophotometry (Solarbio, Beijing, China). Adenosine triphosphate (ATP) levels in the liver were determined by high performance liquid chromatography (HPLC) with a Sepax Bio-C18 (4.6 mm i.d. × 250 mm, 5 μm, 200 A) column and a UV detector at a wave length of 254 nm (BW 16 nm) [77].

### 4.5. Statistical Analysis

All values were analyzed with GraphPad Prism software (La Jolla, CA, USA) and expressed as mean ± standard error of the mean (SEM). Statistical analysis of the western blot parameters and comparisons between groups were performed using a one-way analysis of variance (ANOVA). Statistical significance was assumed at *p* < 0.05.

## 5. Conclusions

In conclusion, our findings show that acute cold stress in mice was found to increase O-GlcNAc modification levels, which may have resulted in the decrease of the essential processes of apoptosis and autophagy, promoting cell survival, while altering glycose transport, glycogen synthesis, and glycolysis in the liver.
